# Sequence Analysis of Hypothetical Lysine Exporter Genes of *Rhizobium leguminosarum* bv. *trifolii* from Calamine Old Waste Heaps and Their Evolutionary History

**DOI:** 10.1007/s00284-013-0303-z

**Published:** 2013-01-16

**Authors:** Ewa Oleńska, Wanda Małek

**Affiliations:** 1 Department of Genetics and Evolution, University of Białystok, Białystok, Poland; 2 Department of Genetics and Microbiology, University Maria Curie-Skłodowska, Lublin, Poland

## Abstract

The aim of this study was to identify heavy metal detoxification system in* Rhizobium leguminosarum* bv.* trifolii* isolated from* Trifolium repens* inhabiting old (70–100 years) Zn–Pb waste heaps in Poland by PCR reaction with czcD1 and czcD2 primers. By sequence analysis, four different genotypes of obtained amplicons were identified among eight examined isolates. Their sequence similarity ranged 91–99 %. They indicated the highest sequence identity to the hypothetical lysine exporter gene of* R. leguminosarum* bv.* trifolii* WSM1325 (91–97 %) and 76–81 % sequence similarity to hypothetical lysine exporter genes of* R. leguminosarum* bv.* trifolii* WSM2304 and* R. etli* CFN42 and CIAT652. On phylogenetic tree of obtained amplicons, all four studied* R. leguminosarum* bv.* trifolii* genotypes formed common monophyletic cluster with* R. leguminosarum* bv.* trifolii* WSM1325 at 100 % bootstrap support showing that all four amplicons obtained in PCR with czcD1 and czcD2 primers are fragments of hypothetical lysine exporter gene (lysE). We also suggest that Lys efflux exporter may participate in heavy metal transport out of* R. leguminosarum* bv.* trifolii* cells.

## Introduction

According to extensive human industrial and agricultural activities heavy metal content in soils still grows [23]. Some heavy metals like cobalt, copper, nickel, and zinc in small concentrations are essential to many cellular processes of microorganisms. For instance, cobalt functions as a cofactor of cobalamin and other enzymes like methionine aminopeptidase, nitrile bromopeptidase, or lysine-2,3-aminomutase [11]; copper because of its convenient redox potential is essential as a prosthetic group of enzymes involved in the reduction of nitrate and nitrous oxides [24], whereas nickel is utilized for function of metal-dependent urease, carbon monoxide hydrogenase, glyoxylase, acetyl-coenzyme A decarbonylase, or superoxide dismutase [13]. Behind their essential character, heavy metals are usually cytotoxic even in small quantities, they persist in the environment and are risky to all living organisms [1, 7]. Heavy metals may reduce biodiversity, significantly limit reproduction and growth as well as activity of bacteria i.e., nitrogen fixation in diazotrophs [17]. Some organisms evolved several mechanisms of resistance to cope with metals toxicity and they are tolerant to heavy metals. These mechanisms make bacteria able to survive by detoxification mechanisms developed in direct response to the metal [3, 6]. It is thought that abilities of bacteria to tolerate toxic heavy metals might have arose due to their long-term co-existence with high heavy metals concentrations in environment shortly after prokaryote life started [9]. There are three types of toxic metals tolerance in bacteria which involve active (ATP-dependent) or passive (ATP-independent) transport of the ionic forms of metals out of the cell, enzymatic detoxification into a less toxic metal forms, and occasionally immobilization of the metal by proteins, peptides, and amino acids [12]. Proteins involved in heavy metal resistance in bacteria are chromosomally or extrachromosomally encoded [4]. So far, some metal-specific resistance pumps have been identified in bacteria i.e., *ars* pump for arsenic (AsIII) and antimony (SbIII), *mer* pump for mercury (HgII), *cop* pump for copper (CuII), *cad* pump for cadmium (CdII) and zinc (ZnII) resistance in Gram-positive bacteria or *czc* pump for cadmium (CdII), zinc (ZnII) and cobalt (CoII) resistance in Gram-negative bacteria [9, 21].

Heavy metal contaminated areas, besides of natural origin, most often developed as the result of human activities. For example, calamine (Zn–Pb) waste heaps are highly transformed areas of extremely enormous heavy metals content in the soil, deficient in water and nutrients [25]. Such harsh edaphic conditions make metal contaminated environments highly disadvantageous for survival of various organisms like animals or plants whose biopotential could accelerate restoration of homeostasis of the disturbed places, for example through the accumulation of metals in its tissues [2]. Rhizobia are the group of Gram-negative proteobacteria that make symbiotic relationship with leguminous plants. For example, *Rhizobium leguminosarum* bv. *trifolii* forms symbiotic association with *Trifolium repens* (white clover) roots, and as a result nodules are formed [30]. In nodules, rhizobia fix gas nitrogen from atmosphere and convert it into ammonia, a form highly available to a host–plant improving growth of a plant and it is of great importance particularly on nutrients deficient soils of old (70–100 years) zinc–lead waste heaps like Bolesław and Olkusz ones in southern Poland. Organisms surviving in those highly difficult conditions usually are adapted to toxic heavy metals concentrations and possess genetically determined mechanisms of tolerance. In Gram-negative bacteria the ATP-independent efflux pump Czc occurs which transports zinc and cadmium ions out of the cell [15]. So far there is scarce knowledge about genetically determined mechanisms of ATP-independent heavy metal resistance in *R. leguminosarum* bv. *trifolii* which colonizes highly contaminated natural habitats [19]. The long-ago (70–100 years) created Zn–Pb waste heaps as Bolesław and Olkusz are good laboratory field for studying the genetically determined heavy metals resistance mechanisms in bacteria. Therefore, the aim of this study was to identify heavy metal detoxification system in *R. leguminosarum* bv. *trifolii* isolated from *T. repens* inhabiting old (70–100 years) calamine waste heaps in Poland by PCR.

## Materials and Methods

### Study Area

The study was performed on metalliferous sites localized in southern Poland. As a study area in present work the old (about 70–100 years old) Zn–Pb waste heaps in Bolesław (50°17′N 19°29′E) and Olkusz (50°17′N 19°4′E) were chosen. They are inhabited by metallicollous plant populations. Average content of metals in soil of waste heaps is: 40,000 mg Zn × kg^−1^, 3,000 mg Pb × kg^−1^, 170 mg Cd × kg^−1^, 36 mg Ni × kg^−1^, and 43 mg Tl × kg^−1^ d. wt. [25], whereas the highest permissible content of metals in arable lands is: 100–300 mg Zn × kg^−1^, 50–100 mg Pb × kg^−1^, and 0.75–1.50 mg Cd × kg^−1^ d. wt. (Decree of Polish Minister of Agriculture and Rural Development 2002, 37, 334 §1).

### Sampling and Bacterial Isolation

*Rhizobium* *leguminosarum* bv. *trifolii* strains were isolated from the white clover root nodules collected in the heavy metals polluted fields. Bacterial isolates were obtained from six *T. repens* host–plants (three per one site). White clover is a clonal perennial; therefore, as separate genetic individuals they were regarded as plants with distance of 2 meters from each other. Ten nodules collected from roots of every genetic individual were sterilized, crushed, and streaked on 79CA [28]. Bacterial growth was evident after 2 days of incubation at 28 °C. Single colonies were obtained by dilution method. Isolated rhizobia were confirmed for nodulation ability of common white clover plants by their inoculation in laboratory conditions. *T. repens* seeds at first were surface sterilized and subsequently germinated in darkness [28]. The surface sterilization of seeds was performed by treatment with HgCl_2_ (0.1 %; v/v) for 3 min, washing three times with sterile water for 5 min, once with ethanol (70 %; v/v) for 3 min and three times in sterile water for 5 min. After that seeds were kept in sterile water for 3 h for the swelling, and thereafter transferred into plates with water–agar medium and incubated in 28 °C. Two-day-old seedlings were transferred into glass tubes with Hoagland medium [8]. Seedlings were inoculated with nodule isolates directly onto the roots and cultivated in greenhouse for 6 weeks at 19–23 °C with 12 h of light per 24 h cycle for testing of the ability of forming the nodules which was estimated due to number and color of nodules and the size and color of plants. As controls non-inoculated plants were used. For further analysis in total 20 isolates of *R.* *leguminosarum* bv. *trifolii* were used.

### Isolation of DNA and Analysis of Amplified Fragments

#### Isolation of DNA

For genomic DNA isolation, bacterial isolates were grown on orbital shaker (190 rpm) for 24 h at 28 °C in 5 mL liquid 79CA medium [28] and after that, bacterial cultures were transferred into 25 mL liquid 79CA medium and still incubated in shaker during 72 h at 28 °C. The purity of the cultures was measured after spreading 100 μL of bacteria into 79CA agar plates. The extraction and purification of genomic DNA was performed with, the usage of guanidine thiocyanate according to the method of Pitcher et al. [18]. The cultures were centrifuged at 20,000×*g* for 15 min and pellets were resuspensed in 200 μL TE buffer (10 mM Tris–HCl, 1 mM × L^−1^ EDTA, pH = 8). Cell suspensions were lysed with 1 mL GES reagent (5 M guanidine thiocyanate, 100 mM EDTA, and 0.5 % v/v sarkosyl), vortexed and incubated in room temperature for 5–10 min. The lysates were cooled on ice and 0.5 mL cold 7.5 M ammonium acetate was added with mixing for 10 min. After that 1 mL of chloroform and isoamyl alcohol (24:1) mixture was added. The phases were mixed and centrifuged at 20,000×*g* for 15 min. Supernatants were transferred into Eppendorf tubes and 540 μL of cold isoamyl alcohol was added. The samples were manually mixed for 1 min and cooled in −20 °C for 30 min. After that samples were centrifuged at 20,000×*g* for 15 min. Pellets of DNA were washed three times in 100 μL 70 % ethanol and centrifuged at 20,000×*g* for 5 min. The obtained DNA was dried under vacuum for 1–5 min, resuspensed in 100 μL of sterile deionized water, and redissolved overnight at 4 °C. RNA was removed by addition of 2 μL RNAse per sample (A&A Biotechnology). DNA concentration and its purity was measured spectrophotometrically at λ = 260 and 280 nm in SmartSpec™3000 BioRad.

#### Analysis of Amplified Sequence Fragments

For *czcD* gene amplification in PCR reaction DNA with final concentration of 50 ng DNA, 30 pmol of the oligonucleotide sequences as primers czcD1 (5′-AACCAGATCTCGCGCGAGAAC-3′) and czcD2 (5′-CGGCAACACCAGTAGGGTCAG-3′) [1], PCR DIG Probe Synthesis Kit (Roche) consisting of PCR DIG Probe Synthesis Mix, PCR Buffer with MgCl_2_, and enzyme in a total reaction volume of 50 μL were used. Amplifications were carried out in an Applied Biosystems ThermalCycler model 2720 with the following temperature profile: initial denaturation at 94 °C for 30 s, 5 cycles of denaturation at 94 °C for 30 s, annealing at 54 °C for 30 s, and extension at 72 °C for 1 min, 30 cycles of denaturation at 94 °C for 30 s, annealing at 64 °C for 30 s, extension at 72 °C for 1 min and final extension at 72 °C for 5 min [16]. DNA amplicons were subjected to 1 % agarose gel electrophoresis in 1× concentrated TBE buffer for 1 h in 100 V with GeneRuler™100 bp DNA Ladder #SMO243 80–1,031 bp (Fermentas) as a marker. Amplified DNA was purified with “CleanUp kit” (A&A Biotechnology). Sequencing PCR was performed by dideoxynucleotide chain-termination method (Big Dye Terminator CycleSequencing Kit Applied Biosystems) in reaction conditions as follows: initial denaturation at 96 °C for 1 min and 25 cycles of denaturation at 96 °C for 10 s, annealing at 50 °C for 5 s and extension at 60 °C for 2 min. Purification of DNA after sequencing reaction was performed with ExTerminator Kit (A&A Biotechnology) according to protocol, and sequenced with ABI Prism 3130 capillary sequencer (Applied Biosystems).

#### Phylogenetic Analysis

Multiple sequence alignments were performed with ClustalX [27] and corrected manually with the usage of GeneDoc software [14]. The characteristic of genotypes was performed using MEGA4 and Arlequin programs. Alignments were compared with sequences deposed in GenBank NCBI with the usage BLAST algorithm. The similarity of nucleotide sequences (%) was calculated with GeneDoc program. Phylogenetic trees were constructed using the Neighbor-Joining (NJ) method. The phylogenetic distances were estimated with the Kimura 2-parameter (K2P) model [10] using MEGA4 program [26]. To determine the degree of statistical support for branches on phylogram, 100 bootstrap replicates of the data were analyzed.

## Results and Discussion

The proper function of heavy metal tolerance mechanisms improves growth of organisms living at difficult conditions particularly on high heavy metal contaminated areas which are localized for example in Poland in Olkusz region. Czc system is one of heavy metal tolerance pathways which have been evolved in bacteria. 20 isolates of *Rhizobium leguminosarum* bv. *trifolii* obtained from nodules of wild-growing *T. repens* were examined for the presence and phylogeny of the metal/H^+^ antiporter *czcD* gene whose protein product might be involved in zinc and cadmium resistance of studied bacteria isolated from *Trifolium repens* growing in old (70–100 years) Bolesław and Olkusz calamine (Zn–Pb) waste heaps. Using PCR with czcD1 and czcD2 primers DNA fragments of 623-bp in length were amplified in eight of them. Four isolates were obtained from Bolesław waste-heap area (7.1, 7.2, 7.3, 7.6) and other (8.1, 8.2, 8.3, 8.5) originated from Olkusz site. The analysis of obtained amplicon sequences allowed to distinguish four genotypes among studied *R. leguminosarum* bv. *trifolii* isolates: genotype 1 (KC 145730) comprised 7.1, 7.2, 7.6 isolates, genotype 2 (KC 145731) included 7.3 isolate, genotype 3 (KC 145732) was composed of 8.1 isolate, and genotype 4 (KC 145733) comprised 8.2, 8.3, 8.5 isolates. The comparative sequence analysis of 623-bp DNA fragments obtained in the PCR reaction of four identified genotypes revealed 49 polymorphic sites. Among 50 substitutions, 29 transitions and 21 transversions were noted. Polymorphic loci of amplified DNA fragments detected in symbionts of white clover growing in old waste heaps are presented in Table 1.

**Table 1:** Polymorphic loci of sequence fragments amplified with czcD1 and czcD2 primers in four genotypes of *R. leguminosarum* bv. *trifolii* isolated from nodules of *T. repens* collected in Bolesław and Olkusz Zn–Pb waste heaps

Genotype	Number of polymorphic loci																								
	58	61	64	103	118	140	145	154	155	172	179	204	205	207	226	233	235	241	245	247	259	277	294	295	331
1	G	T	C	C	A	G	G	G	C	A	T	A	G	C	A	G	T	G	A	A	G	A	T	A	C
2				T						G															
3	T	C	G		G		A	A	G	A	C	G	C	A	T	A	C	A	G	C	C	G	C	G	T
4						C									A										

Genotype	Number of polymorphic loci																							
	335	337	343	355	361	371	375	383	388	389	399	409	414	418	466	502	508	535	544	553	556	566	567	574
1	C	G	G	C	T	G	G	C	A	G	G	T	G	G	C	A	A	G	G	A	G	A	T	T
2																								
3	A	C	A	G	C	C	A	A	G	A	C	G	A	T	G	G	G	A	C	C	C	G	C	C
4														A										

The comparison of 348-bp long amplified sequences of examined genotypes with those of reference rhizobia (GenBank database) due to BLAST algorithm revealed the highest − 97 % sequence identity with the conserved hypothetical lysine exporter protein gene (LysE/YGGA) of *R. leguminosarum* bv. *trifolii* WSM1325 strain (CP 001622). The LysE/YGGA protein product is an unidirectional efflux transporter with proton motive force and belongs to superfamily of proteins involved in transport of amino acids and heavy metals [18]. Sequences amplified in PCR reaction with the czcD1 and czcD2 primers also revealed 76–81 % identity with lysine exporter gene sequence of *R. leguminosarum* bv*. trifolii* WSM2304 (CP 001191), 79 % identity with amino acid efflux protein of *R. etli* CIAT 652 (CP 001074) and *R. etli* CFN 42 (CP 000133) (Table 2). Product of the l*ysE* gene possesses similar structure to CzcA protein, a member of the Czc system and it is possible that both of them may perform similar function. According to Diels et al. [5], ATP-dependent transporters possessing six hydrophobic domains and 12-transmembrane helix chemiosmotic transporters have the same overall structural organization and use the same class of protein in their export pathway. LysE exporter exhibits six hydrophobic domains which in a dimer form could correspond to 12-transmembrane helical spanners of the CzcA protein which arose possibly due to tandem intragenic duplication [22, 29]. Therefore, it cannot be excluded that in *R. leguminosarum* bv. *trifolii* obtained from nodules of white clover which inhabited 70–100 years calamine waste heaps, LysE/YGGA transporter functions as heavy metals exporter.

**Table 2:** Nucleotide similarity [%] of amplified with czcD1 and czcD2 primers sequence fragments in analyzed genotypes *R. leguminosarum* bv. *trifolii* received from *T. repens* nodules collected in Bolesław and Olkusz Zn–Pb waste heaps and reference rhizobia strains (GenBank database)

	Genotype 1	Genotype 2	Genotype 3	Genotype 4
Genotype 1	100	99	91	91
Genotype 2	99	100	91	91
Genotype 3	91	91	100	99
Genotype 4	91	91	99	100
*R. leg.* bv. *tri*. WSM1325	97	97	91	91
*R. etli* CFN42	79	79	76	77
*R. leg.* bv. *tri.* WSM2304	81	81	80	80
*R. etli* CIAT652	79	79	79	79

**Fig. 1:**
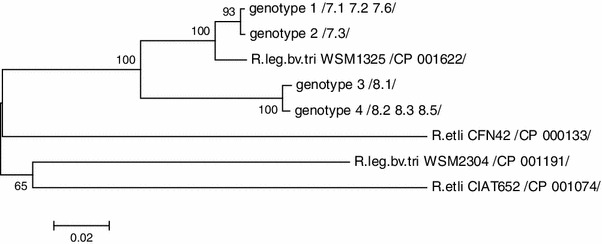
Phylogenetic NJ tree based on 348-bp fragment amplified with czcD1 and D2 primers of four genotypes of *R. leguminosarum* bv. *trifolii* and reference rhizobia strains (GenBank database)

The comparative sequence analysis of DNA amplified with czcD1 and czcD2 primers revealed 91–99 % nucleotide identity of four rhizobium genotypes obtained from root nodules of *T. repens* growing on old (70–100 years) Zn–Pb waste heaps Bolesław and Olkusz (Table 2). Amplicons czcD1-czcD2 of *T. repens* nodule isolates from southern Poland Bolesław calamine waste heap (genotypes 1 and 2) differed from Olkusz waste-heap rhizobium isolates (genotypes 3 and 4) in 9 % nucleotides. The relationship of these sequences with those of reference rhizobia is presented on Fig. 1. The phylogenetic NJ tree of *lysE* gene constructed on the basis of the degree of the nucleotide similarity by Kimura 2-parameter (K2P) model showed that studied rhizobium genotypes are closely related to *R. leguminosarum* bv.* trifolii* strain WSM1325, which was isolated from a nodule recovered from roots of an annual clover plant growing near Livadi beach on the Greek Cyclades island in Serifos [20]. All these genotypes formed common cluster with 100 % bootstrap support suggesting that they are of monophyletic origin. *R. leguminosarum* bv. *trifolii* strain WSM2304 and two *R. etli* strains (CFN42 and CIAT653) with analyzed gene sequence identities, respectively, 80–81 and 76–79 %, forming separate lineages with bootstrap support lower than 70 % were included into analysis.

## Appendix

Nucleotide sequence comparison 348-bp fragment amplified with czcD1 and D2 primers of four *R. leguminosarum* bv. *trifolii* genotypes received from *T. repens* nodules collected in Bolesław and Olkusz Pb–Zn waste heap with rhizobia reference strains (GenBank database). Color marking: black—100 % sequence similarity, dark gray—80–99 % sequence similarity, light gray—60–79 % sequence similarity.
